# Temporal complexity of LVV-hemorphin-7 allosterism at the angiotensin II type 1 receptor assessed using entropy-based approaches

**DOI:** 10.1016/j.csbj.2025.10.024

**Published:** 2025-10-14

**Authors:** H.F. Jelinek, M.P. Johnson, F.B. Khan, M. Elgendi, M.A. Ayoub

**Affiliations:** a Department of Clinical Sciences, College of Medicine and Health Sciences, Khalifa University of Science and Technology, Abu Dhabi 127788, UAE; b Healthcare Engineering Innovation Group, Khalifa University of Science and Technology, Abu Dhabi 127788, UAE; c Department of Public Health and Epidemiology, College of Medicine and Health Sciences, Khalifa University of Science and Technology, Abu Dhabi 127788, UAE; d Department of Biological Sciences, College of Medicine and Health Sciences, Khalifa University of Science and Technology, Abu Dhabi 127788, UAE; e Department of Biomedical Engineering and Biotechnology, College of Medicine and Health Sciences, Khalifa University of Science and Technology, Abu Dhabi 127788, UAE; f Center for Biotechnology, Khalifa University of Science and Technology, Abu Dhabi 127788, UAE

**Keywords:** Allosteric modulation, Molecular pharmacology, Multiscale Rényi entropy, Sample, Entropy, G protein-coupled receptors (GPCRs)

## Abstract

Allosteric modulation constitutes an interesting aspect of the molecular pharmacology of hormone receptors and enzymes with implications in basic research and drug discovery. The modulation of the angiotensin II type 1 receptor by the endogenous peptide, LVV-hemorphin 7 (LVV-H7), is an example that was recently reported using various *in vitro* pharmacological and biochemical approaches as well as *in silico* analysis. In this study, we used real-time biosensor data using BRET technology and applied sample entropy and multiscale Rényi entropy to measure the effect of LVV-H7 on receptor activity over time. LVV-H7 increased signaling complexity when used alone and stabilized receptor activity when combined with angiotensin II. These effects were different for the Gαq and β-arrestin signaling pathways. The results indicate that LVV-H7 functions to fine-tunes receptor behavior in a dynamic and pathway-specific manner. This highlights the value of entropy-based tools for tracking changes in cell signaling and further exploring hormone receptor pharmacology and signaling with potential applications in drug design and analysis. The study also suggests that endogenous peptides such as LVV-H7 could offer new ways to modulate the angiotensin II receptor for therapeutic benefit.

## Highlights

• Our findings link statistical physics of entropy to receptor pharmacology and drug design.
• The entropy-based analysis identified time-varying complexity in GPCR signaling.
• LVV-Hemorphin-7 has the potential to modulate the AT1 receptor by dynamic and pathway-specific mechanisms.
• Sample entropy and multiscale Rényi entropy describe configurational transitions of the receptor specific to ligand binding.
• LVV-H7 enhances AngII binding, stabilizes receptor dynamics and indicates change in receptor configurational dynamics.

## Graphical Abstract

**Figure:**
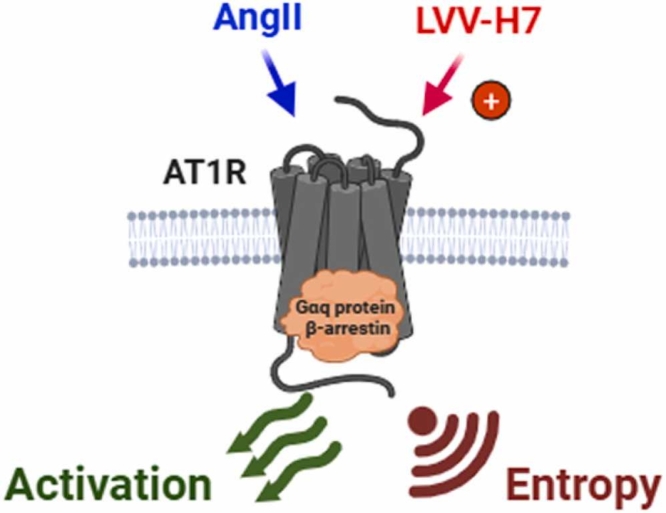

## 1 Introduction

Peptide allosteric modulation has recently been recognized as an alternative to medical intervention based on innovative drug design and discovery due to limited opportunities for further drug development on orthostatic sites [1], [2]. Allosteric regulation in proteins functions through multiple existing pathways associated with the protein structure and its changes. The dominant pathways depend on the protein structure, binding events, modifications, environmental conditions, and perturbations. Perturbations such as peptide or drug binding to the protein shift the balance and activity between existing pathways, altering the distribution of protein states and leading to different functional outcomes. Allosteric responses do not require major conformational changes but result from dynamic shifts within the inherent signaling network of the protein [3].

Allosterism at G protein-coupled receptors (GPCRs) provide an opportunity to investigate ligands other than endogenous orthosteric agonists that modulate receptor activity through binding at spatially separate sites. Allosteric modulators do not have intrinsic agonist activity but enhance GPCR signaling in the presence of their orthosteric ligands, indicating a cooperative allosteric interaction by influencing conformational changes of the receptor or dynamic shifts within the inherent signaling network of the protein, which modifies the efficacy, potency, and/or pathway preference of the orthosteric ligands [4]. This has led to an expansion in the field of drug discovery by highlighting alternative therapeutic approaches based on allosteric modulation of GPCRs, with many molecules that were identified as either positive or negative modulators of GPCRs [2].

Biased signaling is another important concept in GPCR pharmacology with potential therapeutic applications [5]. It describes the ability of a single pharmacologically active molecule to specifically activate one subset of the GPCR downstream pathway over others. In the simple, classical scenario, a ligand binding to a GPCR leads to its activation, which then triggers G protein and arrestin pathways, resulting in various intracellular responses. Any ligand that activates both G protein- and arrestin-dependent pathways is a balanced agonist. However, GPCR pharmacology has advanced toward a more detailed and precise approach, where a class of ligands can selectively activate (agonists) or block (antagonists) either G protein-dependent or arrestin-dependent pathways, but not both at the same time. These ligands are often classified as G protein-biased or arrestin-biased. The concepts of allostery and bias can also overlap, as allosteric modulators may exhibit biased actions on GPCRs [6], [7]. Among GPCRs, the angiotensin II type 1 receptor (AT1R) provides a prototype for investigating allosteric modulation, with recent studies showing that allosteric regulation of AT1R dynamically influences receptor coupling to G proteins or β-arrestins through changes in conformational entropy [8], [9], [10]. This specific receptor signaling architecture and conformational dynamics may be important in therapeutic applications related to the pathophysiological circumstances associated with the renin-angiotensin system, where AT1R is known to play a pivotal role [11].

Among the diverse classes of endogenous and exogenous GPCR ligands, hemorphins, which are atypical opioid peptides derived from proteolytic cleavage of hemoglobin, are useful for their pleiotropic interactions with various GPCRs, including opioid, oxytocin, bombesin, and angiotensin receptors [12]. Specifically, LVV-hemorphin-7 (Leu-Val-Val-Tyr-Pro-Trp-Thr-Gln-Arg-Phe; LVV-H7) is a bioactive decapeptide derived from the N-terminal region of hemoglobin β-chain. LVV-hemorphin-7 exhibits ligand-directed signaling and can thus function as either an orthosteric agonist or an allosteric modulator, depending on receptor subtype and physiological context [13]. Recent molecular and in vitro studies investigating dose-response curve characteristics have demonstrated that the allosteric action of LVV-H7, which can bind and positively modulate AT1R, leading to stabilization of the receptor conformational changes [14]. This binding influences AT1R downstream signaling, which may have therapeutic potential for modulating maladaptive responses in hypertensive or inflammatory pathophysiology by potentially correcting dysregulation in diseases such as hypertension [15]. Other behavioral effects of LVV-H7 have been reported to be mediated through the oxytocin receptor, which further broadens its GPCR target spectrum, including socio-affective neuromodulation [16].

Receptor–ligand interactions are not solely governed by static affinity or binding energy but are influenced by the entropy of the ligand and receptor conformational changes. In GPCRs, ligand recognition is an entropy-driven process in which receptor plasticity and configurational transitions play central roles in determining downstream signaling bias [17], [18], [19]. Ligands such as LVV-H7 may induce receptor conformations that maintain flexibility across multiple microstates, allowing selective recruitment of G protein or β-arrestin in a context-dependent manner [8], [20]. These conformational changes can be identified using diverse approaches and models.

Time-aligned similarity assumes that dynamic processes unfold in a temporally coordinated, reproducible fashion across trials or conditions. Ligand-induced receptor states shift the amplitude or timing of the reconfiguration time series, thereby altering the temporal characteristics across multiple scales. This introduces a breakdown of ergodicity and introduces memory and aging effects into the system dynamics, which indicate complex adaptive behavior [21]. Hence, the current model is based on a non-ergodic renewal process such as fractional kinetics [21]. When applying this model to GPCR signaling, ligands such as LVV-H7 influence receptor states that alter downstream amplitude or kinetics and reconfigure the temporal characteristics of the signaling cascade that entropy measures can analyze [22]. The non-ergodic renewal framework [21], where the system exhibits heavy-tailed waiting time distributions between transitions that are associated with the kinetic diversity of receptor states [22], wherein GPCRs undergo heterogeneous switching between active conformational substates, resulting temporally irregular activation patterns similar to signaling bursts in GPCR activity.

To capture receptor dynamics, entropy-based approaches such as sample entropy (SampEn) and multiscale Rényi entropy (msRE) can quantify the underlying statistical structure and complexity of the temporal dynamics of signaling outputs characterized by temporally irregular activation patterns before returning [23], [24], [25], [26]. SampEn provides a measure of signal regularity by detecting recurrent patterns in receptor activation time series [27]. While SampEn is suitable for detecting irregular signal bursts (e.g., β-arrestin pulses), it does not account for scale-dependent configurational changes that may emerge through biophysical hierarchies such as receptor clustering, internalization, or cAMP oscillations. MsRE analysis addresses this by analyzing the entropy of the time series at various temporal scales [28], [29]. This approach offers improved discrimination of complexity across conditions, such as AngII binding alone versus AngII with LVV-H7 when applied to GPCR systems. msRE can track amplitude changes in signaling, as well as the temporal complexity and adaptability of receptor signaling. Peptides such as LVV-H7 can induce long waiting-time intervals between β-arrestin-mediated signaling bursts, which could signify non-Markovian receptor reconfiguration and correlate to a state of heightened adaptability or constraint, depending on the physiological context. In addition, AngII-induced AT1R activation may yield stereotyped, low-entropy fluctuations dominated by canonical Gq signaling. Hence, LVV-H7 modulation is expected to produce increased fluctuations characterized by entropy and scale-dependent variability, associated with non-Markovian transitions, signal bursts, and reorganization of the intracellular signaling architecture. Entropy measures may provide additional functional information of ligand bias, giving rise to a dynamic biomarker for therapeutic efficacy that quantifies these deviations from ordinary statistical behavior [30], [31], [32].

In the present study, SampEn and msRE were used to investigate and quantify how LVV-H7 alters the adaptability of GPCR systems by shifting response amplitudes or latencies, and by reconfiguring the statistical structure of signaling over time. This approach to the analysis of allosteric modulation highlights that allosteric modulation is not a static property of ligand-receptor interaction, but a dynamic modulation of receptor system complexity, and provides a synthesis between molecular pharmacology and nonequilibrium statistical physics [33]. Applying SampEn and msRE to quantify receptor adaptability is supported by various structure-based mechanical models of allosteric mechanisms based on thermodynamic frameworks as well as computational studies that link GPCR structural networks to signaling bias [34], [35], [36].

**Fig. 1:**
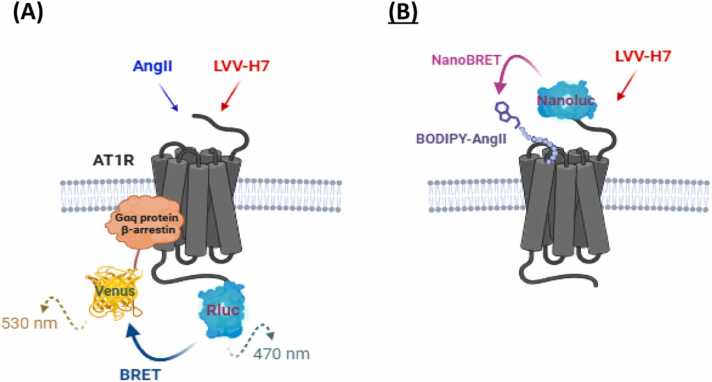
The principle of BRET assays used for AT1R-Gαq protein/β-arrestin interactions (A) and AngII binding to AT1R (B) in live HEK293 cells.

By comparing SampEn and msRE profiles, dynamic biomarkers can be extracted that reflect the temporal reconfiguration of signaling architectures under ligand binding, assessed by real-time kinetic analysis using bioluminescence energy transfer (BRET) technology (Fig. 1). This provides a mechanistic perspective and a quantitative discriminator for classification of receptor configurational adaptation.

Fig. 1 illustrates the experimental design of BRET biosensor assays used to track real-time AT1R signaling via either Gαq or β-arrestin association to AT1R. The diagram illustrates the binding of AngII to AT1R, in the absence or presence of LVV-H7, resulting in downstream G protein or arrestin-mediated responses. For this, Rluc (Renilla luciferase) is a bioluminescent enzyme, and YFP (Yellow Fluorescent Protein) is a genetically encoded fluorescent tag, which are used in BRET assays, as illustrated in Fig. 1A. When the two proteins come into proximity upon receptor activation, energy transfer occurs between Rluc and YFP, which is measured in real-timekinetics.

The present study investigated how entropy-based analysis characterizes LVV-H7 alterations in the temporal dynamics and adaptability of receptor signaling. The results link structural modulation with time-dependent signaling patterns, providing novel insights into how allosteric ligands may influence GPCR function other than traditional binding kinetics.

## 2 Methods

### 2.1 Cell culture and transfection

HEK293 cells were maintained at 37°C, 5 % CO2 in complete medium (Dulbecco’s modified Eagle’s medium (DMEM) containing 0.3 mg/ml glutamine, 100 IU/ml penicillin, and 100 µg/ml streptomycin) supplemented with 10 % fetal calf serum. Transient transfections for BRET assays were carried out in 96-well plates using, per well, 0.5 μl of Lipofectamine 2000 and the appropriate mix of coding plasmids; 25 ng of C-terminally Rluc-fused AT1R with 50 ng of Venus-tagged Gαq or yPET-tagged β-arrestin 2, all in 50 μl total per well of serum free-DMEM. All the experiments were conducted 48 h post-transfection.

### 2.2 Real-time BRET assays

Cells co-expressing AT1R-Rluc and Venus-Gαq/yPET-β-arrestin 2 were first seeded in 96-well white plates, washed with 100 μl/well of phosphate-buffered saline (PBS), and resuspended in 30 μl/well of PBS. BRET measurements were then initiated at 37°C using the Biotek Synergy microplate reader (Agilent Technologies, Santa Clara, CA, USA), allowing the dual detection of light emission at 470 nm and 530 nm. For this, 10 μl/well of Coelenterazine -H (5 µM) was first added to the cells and light emission was recorded for 5–10 min followed by stimulation of cells with 10 μl/well with the indicated peptides, AngII (10 nM), LVV-H7 (10 μM), or their combination, and light emission measurements up to 30 min. The raw data shown represents the 530 nm/470 nm ratio of light emissions at triplicate points for each measurement time.

### 2.3 Entropy measures

SampEn and msRE were chosen as entropy markers based on previous validation in nonlinear biomedical signal studies [22], [31]. They were consistently applied to results from replicated experiments using identical signal preprocessing and windowing functions. The differences in entropy results related to the AngII, and AngII+LVV-H7 ligand conditions aligned with classical pharmacodynamic findings obtained with BRET, supporting the biological relevance and reproducibility of the findings. Future research could incorporate the creation of surrogate signals and larger datasets to further validate these metrics as functional biomarkers of biased GPCR signaling.

### 2.4 Sample entropy

SampEn is a widely adopted method to quantify the regularity and complexity of time-series data. The analysis was conducted in MATLAB (R2023b, Mathworks) following established procedures [27].

Input signals are univariate time series of the ordinary form. Sample entropy was calculated by counting the number of distances between data points separated by an embedding dimension, m+ 1, that exceeds a set threshold. This is compared to the number of distances greater than the threshold between data points separated in the time series by m+ 1. In this study the embedding dimension, m= 1 was used. Whilst is most common in physiological signals [27], using permits for shorter time series, albeit potentially at a loss of sensitivity [37], [38].

(1) $$\mathrm{SampEn}(m,r,N)=\;-\ln\left(\frac{A}{B}∙\frac{N}{N-2}\right)$$

Where A is the count of vector pairs of length 1 (m) less than or equal to r, and B the count of vector pairs of length 2(m+1) less than or equal to r. An embedding dimension of for sample entropy was chosen due to the short duration of each receptor response time series, which ensures sufficient template matching for reliable estimation in brief signaling epochs [37], [38].

All sample entropy calculations are relative and depend on sample length, embedding dimension, and the matching threshold, r. This study used the typical matching threshold, r=0.2*stdDev. Within each experiment, all triplicates and treatments contained the same sampling rate and total number of data points.

### 2.5 Multiscale Rényi entropy

MsRE extends classical information measures by introducing an order parameter α that controls sensitivity to different parts of a distribution. [39]. For a discrete distribution $p={\left\{p_{i}\right\}}_{i=1}^{n}$, the Rényi entropy of order αis defined as

(2) $$H_{\alpha }\left(p\right)=\frac{1}{1-\alpha }\log\left(\sum_{i=1}^{n}p_{i}^{\alpha }\right).\;0<\alpha <\infty \mathrm{and}\alpha \neq 1$$

And where

(3) $$H\left(p\right)=\underset{\propto \to 1}{\lim}H_{\alpha }\left(p\right)=-\sum_{i=1}^{n}p_{i}\log p_{i}$$

Notable special cases include Hartley entropy (α→0: $H_{0}=\log n$), collision (or “quadratic”) entropy (α=2, which emphasizes larger probabilities more than Shannon), and min-entropy (α→∞: $H_{\infty }=-\log\max_{i}p_{i}$). Higher values of min-entropy indicate a highly uncertain state (no single outcome dominates), whereas lower values indicate that the signal is predictable and concentrated on a single state.Whilst common information theory studies consider positive Rényi entropy, this research follows the recommendation of Cornforth et al., [29] to include negative alpha values, expanding further to calculate Rényi entropy for all integer values of α from −10 to + 10, and α=∞. The inclusion of negative α values in the multiscale Rényi entropy analysis is based on previous research demonstrating that negative α values are useful to identify rare but functionally meaningful signal features. Specifically, Cornforth et al. [29] introduced the corrected multiscale Rényi entropy (CMRE) approach and emphasized that negative α scales enable the detection of weakly represented, low-probability states that are often overlooked by conventional entropy measures. In the current context, such states may correspond to transient or low-occupancy receptor microstates that participate in biased signaling. Negative α effectively shifts focus away from dominant states, which are overemphasized at higher α and highlight the tails of the probability distribution, providing complementary information to detect and explain configurational diversity [40], [41]. This rationale is consistent with other studies applying negative α Rényi or related multifractal spectrum methods in EEG, HRV, and fMRI datasets to capture transient desynchronization, metastable dynamics, and subthreshold perturbations often linked to neural or autonomic flexibility [42]. While the biological interpretation of negative α remains an area of research, the use of negative values is supported by its mathematical role in highlighting entropy contributions from rare events. These are physiologically plausible in GPCR signaling, where multiple allosteric sub-states may transiently occur. Thus, the patterns observed at negative scales may reflect genuine functional microstates, especially in the context of allosteric modulation by LVV-H7. Further work with simulated or longer surrogate datasets is needed to validate the current findings further. The probability histogram was calculated using the Gaussian kernel density estimation (KDE) [43], [44]. Bandwidth (σ) was determined by Silverman’s rule [45], and the KDE normalized to sum to 1, to translate the density histogram to probability. Rényi-derived entropy values are normalized by dividing by Hartley entropy, $(H_{0}=\log\left(n\right)$, which limits ${0<H}_{\alpha \geq 1}\leq 1$.

### 2.6 Statistical analysis

The presented results were evaluated for significance using the paired two-tailed student *t*-test.

(4) $$t=\frac{\overset{®}{d}}{s_{d}/\sqrt{n}}$$

Where $\overset{®}{d}$ is the mean difference between paired values, $s_{d}$ the standard deviation of the differences, and n the number of value pairs.

Statistical comparisons between treatment conditions were predefined. Due to the limited number of targeted comparisons and the small sample sizes, formal correction for multiple testing was not applied to avoid inflating Type II errors. Instead, a conservative interpretation method was applied and validated across independent replicates (n ≥ 3). Entropy trends were further assessed for consistency using bootstrap resampling (1000 iterations) to generate 95 % confidence intervals for key differences. All analyses were performed in R and Python using built-in functions and custom scripts.

## 3 Results

### 3.1 Functional coupling of AT1R with Gαq and β-arrestin 2 assessed in real-time and in live cells

**Fig. 2:**
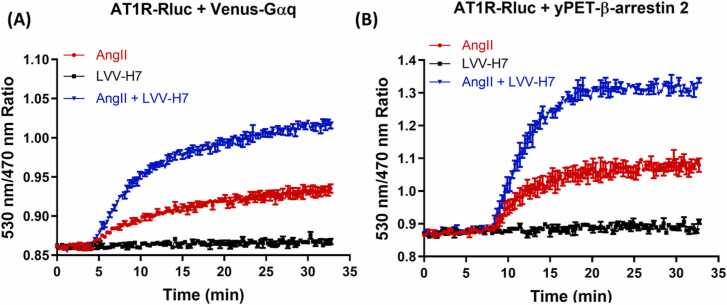
Real-time BRET kinetic results of the association of AT1R with Venus-Gαq (A) and yPET-β-arrestin 2 (B) in HEK293 cells in response to AngII (red curve), LVV-H7 (black curve), or their combination (blue curve). The graphs are representative of 3 independent experiments performed in triplicate (A) or duplicate (B). The data are means ± SEM of the duplicate or triplicate measurements.

As shown previously, LVV-H7 had a positive allosteric effect on AT1R activity and signaling [14], [46]. This was demonstrated in HEK293 cells using the BRET approach to assess the functional coupling of AT1R with its cognate Gαq protein, as well as its interaction with β-arrestin 2 in live cells. Therefore, in this study, we performed real-time BRET kinetics in HEK293 cells co-expressing AT1R-Rluc as a BRET donor with either Venus-Gαq (Fig. 2A) or yPET-β-arrestin 2 (Fig. 2B) as BRET acceptors.

The BRET-based biosensor readouts shown in Figs. 2A and 2B reflect conformational changes and activation dynamics in GPCR-Gα coupling as well as GPCR-Arrestin interaction in real-time and live cells. The curves show that 10 μM of LVV-H7 alone had no agonistic action at AT1R. By contrast, its combination with a sub-saturating dose of AngII (10 nM) strongly potentiated the AngII-mediated signals within both AT1R/Gαq and AT1R/β-arrestin pairs. This occurred in a time-dependent manner with faster kinetics for AT1R/β-arrestin and a saturation reached after 10 min of stimulation. This confirms the synergistic effect, as previously reported [14], [46]. Such BRET kinetic curves describe a typical profile of a positive allosteric interaction between two peptides one (AngII and LVV-H7) at the same GPCR. BRET technology is based on the distance and the orientation, of donor and acceptor sensors within a complex made by two proteins. In this context, AngII-induced BRET signals reflect a specific active conformation of AngII-bound AT1R leading to its functional coupling and association with Gαq and β-arrestin. Thus, any further BRET increase mediated by LVV-H7 suggests the induction and/or the stabilization of different active conformations characterized by better coupling and association between the receptor and the signaling proteins. Indeed, LVV-H7 binds to an allosteric binding site at AT1R, resulting in further movements within the already AngII-bound receptor, which places the BRET donor (Rluc) and acceptors (Venus or yPET within Gαq and β-arrestin, respectively) in a more favorable distance/orientation for BRET increase to occur.

### 3.2 Functional AT1R/Gαq and β-arrestin 2 associations analyzed by entropy-based approaches

**Fig. 3:**
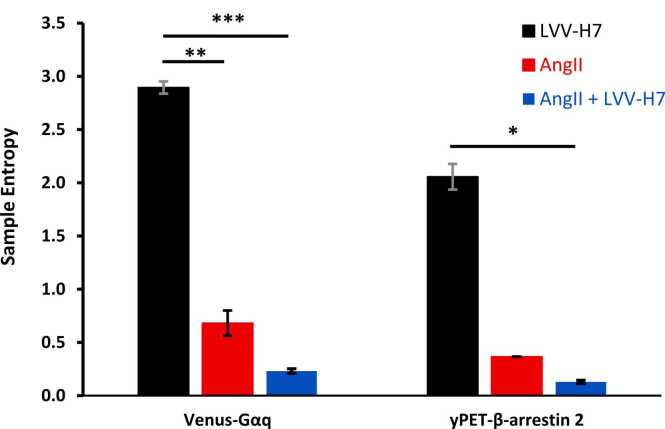
SampEn results for the Venus-Gαq and yPET-β-arrestin 2 real-time BRET assays. The error bars denote the standard error, significance by paired *t*-test where appropriate.

The sample-entropy values for the Venus-Gαq and yPET-β-arrestin 2 real time BRET assay results shown in Fig. 2 are presented below in Fig. 3.

In Fig. 3, the mean values of the SampEn indicate significant differences in AT1R configuration dynamics. AngII binding led to a decrease in SampEn, suggesting more predictability in activity, and is associated with canonical Gq pathway activation. Intermediate receptor entropy, therefore, is a relatively stable configuration dynamic. LVV-H7 binding alone induces alternative conformations with the highest SampEn associated with β-arrestin pathway recruitment. The receptor configuration over the recorded time is the most unstable. The combined condition of AngII+LVV-H7 resulted in the lowest SampEn, which indicates the least degree of receptor configuration changes and suggests a configurational profile that combines features of canonical and alternative signaling pathways with the highest stability. The results confirm the distinct responses of the receptor to AngII, LVV-H7, and a combination of these, indicating significant differences between the mean configurational changes of AT1R depending on ligand binding.

**Fig. 4:**
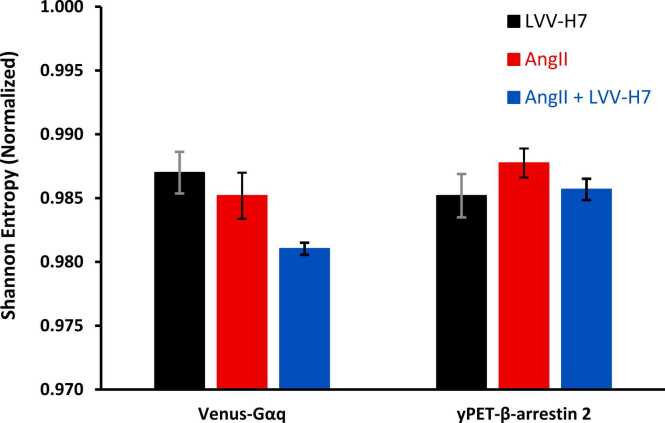
Normalized Shannon entropy (H_1_/H_0_) for Venus-Gαq and yPET-β-arrestin 2 real time BRET assays. The error bars denote the standard error.

Shannon entropy, (H_1_) results are shown in Fig. 4.

The Shannon entropies shown support the idea that AngII+LVV-H7 reduces the conformational entropy of the AT1 receptor better than AngII alone. Shannon entropy did not recognize the reduction in conformational uncertainty that AngII excitation, compared to LVV-H7, demonstrates in Sample entropy values. Entropy values were computed from time-series BRET data across conditions. For Gαq, AngII+LVV-H7 showed lower H₁ (less variability), while β-arrestin signaling exhibited higher entropy with co-treatment, consistent with increased configurational diversity.

**Fig. 5:**
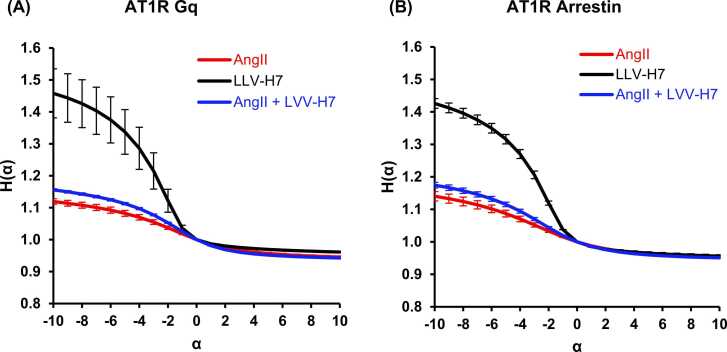
Multiscale Rényi Entropy plots for the Venus-Gαq and yPET-β-arrestin 2 real time BRET assays for assessing scale-dependent signal complexity, error bars denote standard error.

Increasing the number of scales at which the receptor dynamics are analyzed is achieved by applying Rényi entropy analysis for alpha values from −10 to + 10. This demonstrates the lack of distinction between datasets that sample entropy tends to measure (Fig. 5).

Negative α msRE curves for Gαq and β-arrestin show divergent complexity scaling. Gαq responses under AngII+LVV-H7 flatten, while β-arrestin responses increase in multiscale entropy, reflecting greater non-equilibrium complexity.

**Fig. 6:**
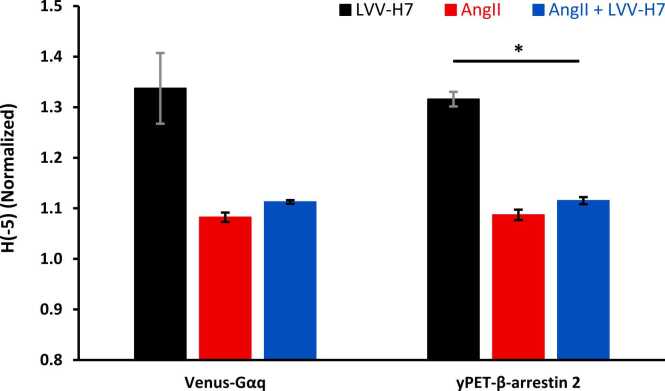
Negative-alpha Rényi (-5) for Venus-Gαq and yPET-β-arrestin experiments. Error bars are standard error.

Fig. 5A and B indicate that the distinction between the datasets for positive-alpha msRE is not as pronounced as that between the datasets for negative-alpha msRE, which are sensitive to rare/low-probability events. The entropy of H_-5_ is plotted in Fig. 6. Note that the same trends continue as α continues toward −10, with improving significance (Fig. 5A and B).

MsRE measures generalized diversity in contrast to SampEn, which measures the conditional probability that patterns in the time series will recur along the time series. Rényi entropy indicates the amount of diversity or complexity inherent in the time series. Both Venus-Gαq and yPET-β-arrestin pathways resulted in similar dynamic relationships by treatment. Negative Rényi Entropies, shown in Fig. 5 and Fig. 6, indicate a greater number of rare, low-probability events for binding with LVV-H7. A reduction in AngII and a slightly lesser reduction following AngII+LVV-H7 indicate lesser dynamics in the channel.

**Fig. 7:**
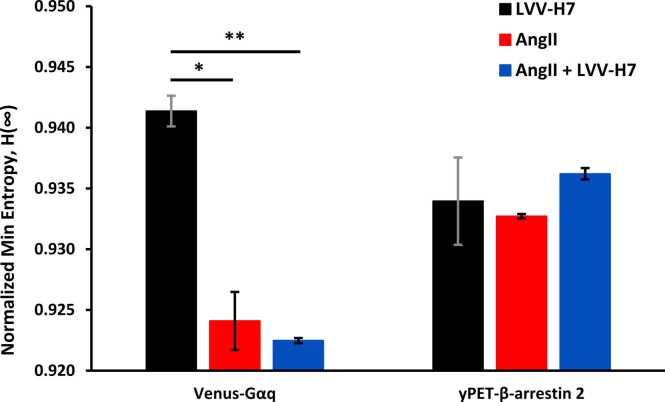
Minimum Rényi entropy (H∞) highlights dominant microstates in signaling. Normalized minimum entropy (H_∞_/H_0_) for both Venus-Gαq and yPET-β-arrestin 2 pathways following stimulation with LVV-H7, AngII, or AngII + LVV-H7. Error bars represent standard error.

This plot depicts the most probable state across conditions. Gαq shows reduced H∞ under AngII+LVV-H7, consistent with stabilized canonical signaling. In contrast, β-arrestin H∞ increases, indicating a lack of dominant microstates and supporting configurational diversification via allosteric bias. The minimum Rényi entropy (H∞) in Fig. 7 quantifies the predictability of the most probable receptor state. A lower H∞ in the Gq pathway suggests signal convergence toward a dominant mode, while increased H∞ in the β-arrestin pathway reflects a broader state ensemble. In comparison, the Shannon entropy (H₁) characteristics shown above in Fig. 4 measure the average uncertainty or diversity of conformational microstates within each signaling pathway. Under AngII and LVV-H7 co-treatment, entropy is reduced in the Gq pathway, indicating stabilization of a dominant receptor-Gq conformation, which is consistent with cooperative allosterism and canonical G protein activation. Whereas, the β-arrestin pathway exhibits a higher Shannon entropy, which reflects greater configurational diversity, and may relate to enhanced functional versatility of arrestin signaling.

The trends observed for SampEn and msRE highlight key features of receptor conformational state dynamics under different ligand conditions. The reduced Shannon entropy and minimum Rényi entropy in the Gq experiment with AngII+LVV-H7 suggest the stabilization of a dominant receptor conformation that promotes canonical G protein signaling. Whereas the higher entropy levels observed in the β-arrestin experiment point to increased configurational flexibility and the engagement of a wider range of receptor states, which may be associated with non-canonical arrestin functions. This divergence in the observed results is consistent with LVV-H7 acting as a conditional allosteric modulator that enhances conformational diversity in a pathway-selective manner and demonstrates how entropy metrics provide a dynamic systems-level perspective of receptor function that agrees with theories of biased signaling and conformational entropy.

### 3.3 Entropy-based analysis of AngII binding and its potentiation in the presence of LVV-H7

Correlation of the BRET data on the functional AT1R/Gαq/β-arrestin 2 with the ligand binding kinetics was also assessed by NanoBRET, as previously reported by Ali et al. [46].

The binding kinetics previously reported by Ali et al., (shown in the original Fig. 3) showed a rapid AngII association to AT1R, demonstrated by the increased gradient, when under the influence of LVV-H7 [46]. The presence of LVV-H7 led to a higher plateau, indicating a more sustained AngII association and binding. The current entropy analyses reveal distinct patterns between the Gq and β-arrestin pathways. The differences found may reflect true pathway-selective modulation or differential sensitivity of Shannon and Rényi entropy to specific features of the signaling time series, which is consistent with the positive effect of LVV-H7 on the functional coupling of AT1R with Gαq and β-arrestin. Further replication and control analyses are required to confirm whether these trends reflect biased allosterism or methodological artifacts. Functional AT1R/Gq and β-arrestin 2 associations were assessed using entropy-based time-series analyses to detect differences in signaling dynamics under LVV-H7 co-treatment, providing insights into pathway-specific modulation not apparent from static amplitude measures.

The allosteric cooperation is also supported by modest increases in maximal AngII binding (Bₘₐₓ) in the presence of LVV-H7, consistent with positive allosteric modulation of AT1R. LVV-H7 does not compete with AngII at the orthosteric site but binds at an intracellular allosteric site, inducing receptor conformational changes that enhance AngII binding affinity or accessibility to active conformers. This may stabilize or enrich a receptor ensemble more receptive to the orthosteric ligand, improving signaling efficiency or amplifying binding responses in BRET assays [47]. Experimental data show increased NanoBRET signals during co-treatment without shifts in static Bₘₐₓ, indicating context-dependent conformational cooperativity rather than simply increasing receptor numbers [46]. Although the binding increase is modest, it is consistent and aligns with structural findings that LVV-H7 promotes deeper ligand insertion and increases hydrogen bonding between AngII and AT1R, emphasizing the functional relevance of allosteric endogenous peptides in modulating GPCR ligand landscapes and supporting computational models of receptor microstate changes prompted by LVV-H7 [48].

**Fig. 8:**
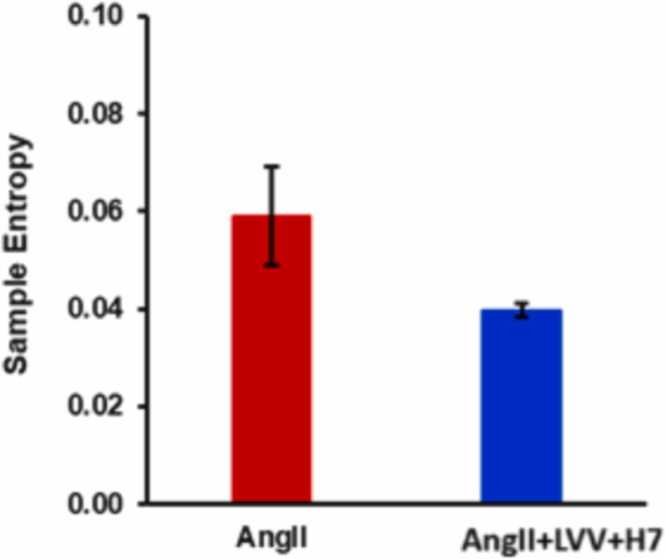
The sample entropies of AngII binding in the absence and presence of LVV-H7. The data are means ± SEM of four independent experiments performed in duplicate measurements.

The SampEn changes are shown as a bar graph in Fig. 8 and quantify the irregularity or complexity of the receptor signaling time series. The decrease in SampEn results indicate that the ATR1 signaling dynamics become more stable, predictable, and less dynamically variable when LVV-H7 is added to AngII. By enforcing a dominant conformational signaling mode, LVV-H7, when coadministered with AngII, stabilizes the AT1R signaling architecture and reduces stochastic fluctuations of the receptor.

**Fig. 9:**
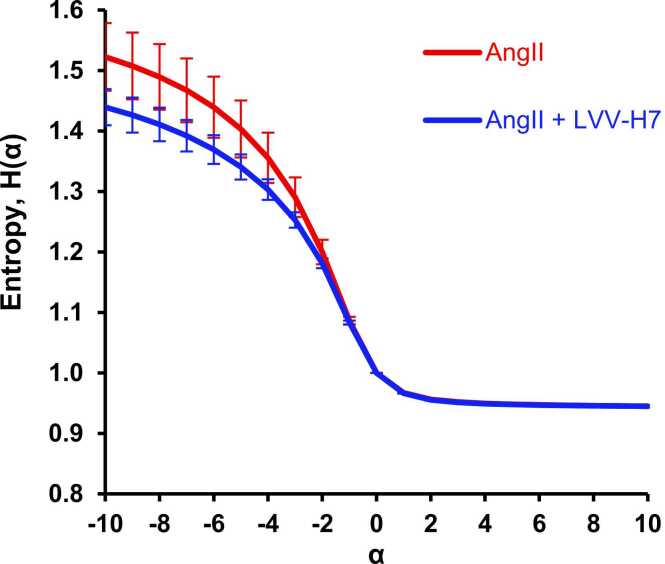
MsRE plot for the binding experiment. The error bars denote the standard error.

MsRE analysis further reinforces the theory that LVV-H7 stabilizes the AT1R architecture, as seen by the decrease in entropy when LVV-H7 was present in Fig. 9, and that conformational changes are still rare events within the time series, demonstrated by entropy differences only being significant at negative-alpha entropies.

## 4 Discussion

To our knowledge, the present study is the first to apply the entropy measures, SampEn and msRE, to assess AT1R receptor activity/configuration dynamics and signaling regularity. These metrics may be useful as proxies for measures of receptor state and specificity dynamics, such as found by combining AngII with LVV-H7 treatment, which reduced entropy in the arrestin pathway, suggesting stabilization of receptor-transducer interactions and a conformational bias. SampEn was used to quantify the irregularity of receptor activation patterns, revealing that LVV-H7 increases signaling complexity, consistent with configurational entropy theories [8], [9], [27], [31], [49]. These two entropy measures are complementary, where H₁ emphasizes global microstate diversity, while H∞ highlights convergence toward dominant states.

LVV-H7 appears to improve the informational capacity of receptor signaling by stabilizing dynamic receptor states, with higher SampEn scores indicating increased flexibility of binding sites, as demonstrated in previous docking and simulation studies [50], [51]. As an allosteric ligand LVV-H7 does not displace AngII or activate signaling on its own. Instead, it conditionally modulates AT1R output, implying allosteric cooperation. This broadens the traditional GPCR modulation view from a one-ligand, one-pathway model to one involving ligand–ligand cooperation that positively influences receptor-mediated responses [51], [52]. Differences in minimum Rényi entropy (H∞) between Gαq and β-arrestin during AngII + LVV-H7 treatment reflect the modulator’s distinct effects on pathway-specific receptor states. In Fig. 7, a reduction in H∞ for Gαq indicates stabilization into a dominant signaling state, while higher H∞ for β-arrestin signifies a wider range of conformations. This aligns with pathway-specific allosteric modulation, where LVV-H7 increases configurational diversity in the β-arrestin pathway without producing symmetrical responses across pathways, supporting models of conformational entropy and GPCR bias discussed in the literature [30], [53], [54]. The allosteric cooperation is also supported by modest increases in maximal AngII binding (Bₘₐₓ) in the presence of LVV-H7, consistent with positive allosteric modulation of AT1R. LVV-H7 does not compete with AngII at the orthosteric site but binds at an intracellular allosteric site, inducing receptor conformational changes that enhance AngII binding affinity or accessibility to active conformers. This may stabilize or enrich a receptor ensemble that is more receptive to the orthosteric ligand, improving signaling efficiency or amplifying binding responses in BRET assays [46], [47]. Experimental data show increased NanoBRET signals during co-treatment without shifts in static Bₘₐₓ, indicating context-dependent conformational cooperativity rather than simply increasing receptor numbers [46]. Although the binding increase is modest, it is consistent. It agrees with structural findings that LVV-H7 promotes deeper ligand insertion and increases hydrogen bonding between AngII and AT1R, emphasizing the functional relevance of allosteric endogenous peptides in modulating GPCR ligand landscapes and supporting computational models of receptor microstate changes prompted by LVV-H7 [48].

The mechanistic link between the entropy measurements and receptor structure can be inferred from insights into crystallography and molecular modeling of AT1R. The high-resolution AT1R structures show how conserved microdomains, such as the DRY motif, transmembrane helix packing, and extracellular loop interfaces, undergo rearrangements during activation [55]. Computational docking and molecular dynamics (MD) studies of LVV-H7 binding have further shown that LVV-H7 associates with intracellular loops 2 and 3 (ICL2/ICL3) and regions of TM3/TM6, stabilizing receptor conformations that favor deeper AngII insertion into the orthosteric pocket [46], [56]. In MD trajectories, the presence of LVV-H7 reduces fluctuations (RMSF) in key activation-related helices and promotes favorable contacts such as a stronger Phe8–Lys199 interaction that suggests a more constrained receptor–ligand interface [56], [57]. From an entropy perspective, these structural stabilizations correspond to lower configurational entropy in canonical Gq signaling, with fewer accessible microstates and higher relative entropy in β-arrestin signaling, with greater conformational flexibility and sampling. The entropy-derived trends found in the current experiments map semi-quantitatively onto known structural transitions of AT1R and predicted modulation by LVV-H7.

The lower minimum Rényi entropy in the Gq pathway and the higher entropy in the β-arrestin pathways indicate divergence between the two pathways, suggesting that AngII + LVV-H7 co-treatment stabilizes canonical G-protein signaling while increasing configurational flexibility in β-arrestin signaling. Reduced entropy in Gq likely reflects a dominant, predictable receptor state linked with vasoconstrictive outcomes, whereas increased entropy in the β-arrestin pathway supports a more diverse signaling ensemble potentially associated with anti-hypertensive or anti-inflammatory pathways. The findings are supported with models of functional selectivity and biased agonism, where endogenous modulators can influence receptor output by stabilizing distinct microstate distributions over time [56], [57].

Entropy-based analyses, such as Sample Entropy and Multiscale Rényi Entropy, provide dynamic, system-level metrics that complement static structural or equilibrium-based thermodynamic assessments. High-resolution structural methods, including X-ray crystallography and cryo-EM, as well as binding affinity assays, provide information on receptor-ligand interactions and conformational states. These methods are constrained to snapshot-based or ensemble-average receptor activity. Entropy measure, as applied here, describes the time-dependent heterogeneity and configurational adaptability of receptor signaling in live-cell contexts [49].

Additionally, entropy-based analysis provides quantitative data on GPCR signaling dynamics and allosteric modulation. SampEn and msRE were lower for the canonical Gq signaling, which may indicate a more predictable receptor activation. β-arrestin with LVV-H7 increased these entropy measures associated with conformation and signal variability. These differences reflect pathway-specific reconfiguration and suggest memory-influenced behaviors missed by standard models. In general, entropy metrics reveal rare events and microstates associated with receptor bias that provide additional biomarkers for functional selectivity. The current results highlight the benefit of applying entropy analysis in profiling drug discovery by clarifying how modulators like LVV-H7 specifically adjust signaling and limit off-target effects [8].

Earlier studies mainly focused on internal receptor motions and conformational entropy using molecular dynamics simulations, NMR spectroscopy, and smFRET, revealing how entropy influences receptor flexibility and coupling efficiency [58], [59], [60], [61]. These studies showed that local entropy fluctuations correspond to functional microstate transitions within GPCR ensembles and that ligand binding shifts these distributions, affecting receptor bias [17], [62]. The current findings build on this by demonstrating that temporal entropy metrics can experimentally capture these configurational transitions in real time, linking structural heterogeneity to functional selectivity. This indicates that entropy could serve as a dynamic biomarker of receptor activation states, connecting computational conformational models with physiological signaling measurements.

Entropy-based approaches quantify the dynamic variability and adaptability of receptor signaling over time. This is particularly interesting when characterizing signaling irregularities, including β-arrestin pulses or G-protein desensitization events [63]. Entropy analysis also detects rare or transient configurational states, especially with negative Rényi entropy, and for tracing how endogenous modulators, such as LVV-H7, reconfigure receptor output in ways that may not directly shift Emax or EC50 but still affect signal encoding fidelity and timing [28], [29], [49], [59].

## 5 Conclusion

Our entropy-based analysis indicates that LVV-H7 modifies the temporal dynamics and configurational complexity of AT1R signaling. Although these findings align with pathway-selective modulation, they are considered a hypothesis rather than conclusive evidence of biased allosterism. Future research involving comprehensive dose–response, structural, and computational modeling will be needed to verify the mechanistic basis of these entropy signatures. This provides a biologically plausible mechanism for tissue-specific or stress-related GPCR modulation by entropy, providing time-resolved insights into these processes. Thus, while earlier studies focused on internal receptor motion and conformational entropy, the current results confirm physiologically and experimentally that entropy can also track pathway-selective signaling outcomes in real-time. This convergence emphasizes the utility of entropy-based metrics not only for structural inference but also for decoding dynamic signaling specificity in live-cell systems. This approach can be further considered for the quantitative and qualitative analysis of hormone receptor pharmacology and signaling with potential applications for drug design, discovery, and characterization. Future research should explore whether LVV-H7 analogs can selectively enhance arrestin coupling without amplifying Gq responses and whether these effects vary across AT1R-expressing tissues.

## CRediT authorship contribution statement

**Johnson Matthew:** Writing – review & editing, Writing – original draft, Software, Methodology, Formal analysis, Conceptualization. **F.B. Khan:** Writing – review & editing, Validation, Investigation, Data curation. **M. Elgendi:** Writing – review & editing, Investigation, Funding acquisition, Conceptualization. **M.A. Ayoub:** Writing – review & editing, Writing – original draft, Validation, Project administration, Investigation, Funding acquisition, Formal analysis, Data curation, Conceptualization. **Jelinek Herbert F:** Writing – review & editing, Writing – original draft, Software, Methodology, Funding acquisition, Formal analysis, Conceptualization.

## Declaration of Generative AI and AI-assisted technologies in the writing process

During the preparation of this work, the author(s) used ChatGPT/Grammarly in order to improve the expression of the written text. After using this tool/service, the author(s) reviewed and edited the content as needed and take full responsibility for the content of the published article.

## Declaration of Competing Interest

The authors declare that they have no known competing financial interests or personal relationships that could have appeared to influence the work reported in this paper.
